# Relationships between Sleep Duration, Timing, Consistency, and Chronotype with Myopia among School-Aged Children

**DOI:** 10.1155/2022/7071801

**Published:** 2022-07-19

**Authors:** Rong Li, Yiting Chen, Anda Zhao, Lili Huang, Zichong Long, Wenhui Kang, Yong Yin, Shilu Tong, Shenghui Li

**Affiliations:** ^1^School of Public Health, Shanghai Jiao Tong University School of Medicine, Shanghai 200025, China; ^2^Shanghai Ninth People's Hospital, Shanghai Jiao Tong University School of Medicine, Shanghai 200011, China; ^3^Shanghai Children's Medical Center, Shanghai Jiao Tong University School of Medicine, Shanghai 200127, China; ^4^School of Public Health, Institute of Environment and Population Health, Anhui Medical University, Hefei 230032, China; ^5^School of Public Health and Social Work, Queensland University of Technology, Brisbane, Australia; ^6^MOE-Shanghai Key Laboratory of Children's Environmental Health, Shanghai Jiao Tong University School of Medicine, Shanghai 200025, China

## Abstract

**Background:**

The role of sleep in childhood myopia has been a research focus; however, the existing evidence is conflicting on sleep duration and timing, and as yet, no studies involve sleep consistency and chronotype. This study is done to make multiple-perspective analyses on the associations between sleep variables and myopia.

**Methods:**

A population-based cross-sectional study was conducted in Shanghai, China, which included 10,142 school-aged children (7–12 years old, 53.2% boys). The Chinese version of the Children's Sleep Habits Questionnaire (CSHQ) was used to assess sleep variables. Propensity score matching was adopted to balance the difference of covariates between nonmyopic and myopic groups. Logistic regression models were implemented to examine the associations between sleep variables and myopia.

**Results:**

Sleep duration and timing, mainly during weekdays, were correlated with myopia in a dose-dependent pattern, in which longer sleep duration was associated with decreased risk of myopia (9-10 hours/day: odds ratio (OR) = 0.87; ≥10 hours/day: OR = 0.77; by comparison with <9 hours/day); later bedtime (9 pm to 9:30 pm: OR = 1.46; 9:30 pm to 10 pm: OR = 1.51; 10 pm and after: OR = 2.08; by comparison with before 9 pm) and later wake-up time (7 am and after: OR = 1.36; by comparison with before 6:30 am) increased the risk (all *P* < 0.05). Moreover, longer weekend catch-up sleep duration and intermediate and evening chronotype were positively correlated with myopia, while social jetlag was associated with a lower odds of myopia. All these findings were also similarly observed in the matching sample.

**Conclusions:**

Multiple dimensions of sleep were involved in childhood myopia. In addition to sleep duration and timing, sleep consistency and chronotype were also strictly related to myopia. More studies are needed to enrich the current evidence, thus further clarifying the association between sleep and childhood myopia.

## 1. Introduction

Sleep is critically important for children's health [1]; however, insufficient sleep and irregular sleep-wake behavior are common worldwide [2]. The United States National Sleep Foundation recommends a sleep duration of 9–11 hours per night for school-aged children (6–13 years old) [3], but data from 12 countries showed that merely 42% of children meet the recommendation [4]. In addition to neural development, metabolic regulation, immune enhancement, and so forth, recent studies noticed that sleep is involved in visual sensitivity [5].

Myopia has been a growing global public health concern in recent decades [6]. It is predicted that the overall number of people with myopia would reach 4.8 billion by 2050 [7]. Data from Asian populations suggested that the rapid increase of myopia occurred during childhood, affecting 80–90% of high school students, among which 10–20% was high myopia [8]. Myopia that happens during middle childhood years is commonly known as school myopia [9], which is usually characterized by a long experience of visual impairment and myopia progression, making children at risk of developing high myopia, even myopic macular degeneration [10].

Given that clinical treatment of myopic pathology is still a big challenge, effective prevention is a top priority. The recent literature revealed that ocular length and other anatomical and physiological features undergo diurnal oscillations and circadian rhythms play a critical role in the regulation of eye growth and refractive error development [11, 12]. Studies in animals found that retinal-specific knockouts of the clock gene can induce myopia in mice, which provided convincing evidence that ocular diurnal rhythm is closely associated with the development of refractive errors [11]. Similarly, ocular and systemic diurnal rhythms were confirmed to be robust in humans [13]. Thus, exploring circadian dysregulation and its association with myopia should be of importance for myopia prevention and intervention.

Growing epidemiological studies have investigated the relationship between sleep and myopia among school-age children and adolescents, while inconsistent findings on the effects of sleep duration and sleep timing on myopia pervade the literature [14–17] and much less attention is devoted to sleep consistency (e.g., weekend catch-up sleep and social jetlag) and chronotype, both of which are important parameters of sleep circadian [18, 19]. Weekend catch-up sleep (CUS) is a common way school-age children recover from sleep loss incurred during weekdays [20, 21], and social jetlag is the discrepancy between social and circadian rhythms [22]. Chronotype refers to the relation of an individual's endogenous circadian clock to the 24-hour day, and it is defined by an individual's timing preferences in engaging in sleep and other activities [23, 24]. In the present study, we conducted a cross-sectional study of 10,142 school-aged children, aiming to systematically examine the relationship between sleep duration, sleep timing, sleep consistency, chronotype, and myopia.

## 2. Materials and Methods

### 2.1. Study Sample

A city-wide cross-sectional survey was conducted during April–June 2019 across Shanghai. To obtain a representative sample, a multistage and multistrata sampling approach were used. Six urban districts and seven suburban/rural districts were randomly selected using a random number method from 16 districts of Shanghai. Then, a total of 17 primary schools were randomly selected. Students in 1st–5th grades in each school were included, and usually, 3-4 classes from each grade were randomly sampled. All sampled children and their parents were told that participation in the survey was voluntary, and written informed consent should be signed to confirm. Among 10,859 children recruited to the study, 10,700 (response rate 98.5%) completed the questionnaires. After the exclusion of those children with hyperopia, amblyopia, strabismus, history of refractive correction surgery, and other eye diseases, 10,142 (94.8%) primary school children aged 7 to 12 years were finally included in this study. The ethical application and the consent procedure of this study were approved by the Ethics Committees of Shanghai Jiao Tong University School of Medicine (SJUPN-201717).

### 2.2. Assessment of Sleep Behaviors

Sleep variables were scaled by Children's Sleep Habits Questionnaire (CSHQ) [25]. In the sampled children, the Cronbach *α* coefficient of the CSHQ questionnaire was 0.91, and test-retest reliability was 0.94. Sleep disorders were defined as total CSHQ scores >41 [25]. In the present study, bedtime and wake-up time were converted into categorical variables (bedtime on weekdays: before 9 pm, 9 pm to 9:30 pm, 9:30 pm to 10 pm, and 10 pm and after; wake-up time on weekdays: before 6 : 30 am, 6 : 30 am to 7 am, and 7 am and after; bedtime on free days: before 10 pm, 10 pm to 10 : 30 pm, 10 : 30 pm to 11 pm, and 11 pm and after; wake-up time on free days: before 7:30 am, 7:30 am to 8 am, and 8 am and after) [15, 16, 26]. Sleep duration on weekdays (SDW) and free days (SDF) were categorized into three groups: < 9 hours, 9–10 hours, and ≥10 hours, based on the recommendations of the United States National Sleep Foundation (9–11 hours per day for 6–13-year-old children) [3]. The average sleep duration was calculated as (5 ×weekday sleep duration + 2 × free day sleep duration)/7. CUS was calculated by sleep duration on free days minus sleep duration on weekdays and grouped into no catch-up (≤0 hours), 0–1 hour, and ≥1 hour [20]. Sleep midpoint was assessed by the bedtime and wake-up time separately for weekdays (MSW) and free days (MSF). Social jetlag was calculated using the absolute difference of the sleep midpoint (in hours) between weekdays and free days. Chronotype was assessed using sleep midpoint on free days corrected for sleep debt on weekdays (MSFsc) [27]. An algorithm proposed by Roenneberg et al. was applied to calculate MSFsc : MSFsc = MSF−0.5 × (SDF−(5×SDW + 2 × SDF)/7), which is only applied if sleep duration was longer on free days than in workdays, and if SDF ≤ SDW, MSFsc = MSF [28]. Tertiles were used to define the chronotypes as the morning, intermediate, and evening [29].

### 2.3. Definition of Myopia

The visual acuity assessment was based on two parent-reported questions. To facilitate students' understanding, myopia was assessed with the following question for parents: “Has your child been diagnosed with myopia?” The response categories were “yes” and “no.” If parents chose “yes,” he or she was asked to indicate the refractive range: low myopia (≤−0.5 to ≥ −3.0 D), moderate myopia (<−3.0 to > −6.0 D), and high myopia (≤−6.0 D). In Shanghai, primary school children have a physical examination every year; if abnormal vision is found, parents are notified that they should accompany their children to the hospital for a series of eye examinations, including an optometry examination. Therefore, most parents who participated in the study would know their children's refractive status. And, the sensitivity and specificity of identifying myopia correctly by self-reporting were reported to be high (0.76 and 0.74, respectively) [30].

### 2.4. Assessment of Covariates

We collected covariates' information covering three aspects: demographic factors, behavioral factors, and dietary factors [31]. Demographic factors were listed as follows: age, gender, body mass index (BMI), fathers' and mothers' education level (below high school degree, high school, or higher), and per capita income (<6000, ≥6000 ¥/month). Behavioral factors contained outdoor time on weekdays/free days (<2, ≥2 hours/day), homework time on weekdays/free days (<3, ≥3 hours/day), TV time (<2, ≥2 hours/day), and digital screen exposure (e.g., computer, tablet, mobile phone, and game console) (<10, ≥10 times/week). Dietary factors included intake frequency of fried food (<1, ≥1time/month), carbonated drinks (<1, ≥1 time/month), and vitamin D (<5, ≥5 times/week).

### 2.5. Statistical Analysis

The statistical description was made by using mean, standard deviation, frequency, and percentage of columns, and the *t*-test and chi-square test were applied to compare differences between groups. A propensity score matching (PSM) method was adopted to adjust and match all covariates between groups (using the R package: “MatchIt”). The strength of PSM lies in taking all covariates along with their interactions as one covariate into account, which could reduce potential selection bias between the nonmyopic group and myopic group [32, 33]. In propensity-score-matched analysis, controls were matched 1 : 1 to cases based on a greedy nearest neighbor matching algorithm on the propensity score with a caliper equaling 0.05. Univariate and multiple logistic regression analyses were performed to determine the odds ratios (ORs) and 95% confidence intervals (CIs) of factors independently associated with children's myopia. A two-tailed *P* value < 0.05 was considered statistically significant. All statistical analyses were conducted using R (version 4.1.1; R Core Team) and Statistical Package for the Social Sciences (SPSS, version 24.0).

## 3. Results

### 3.1. Descriptive Analysis

**Figure 1:**
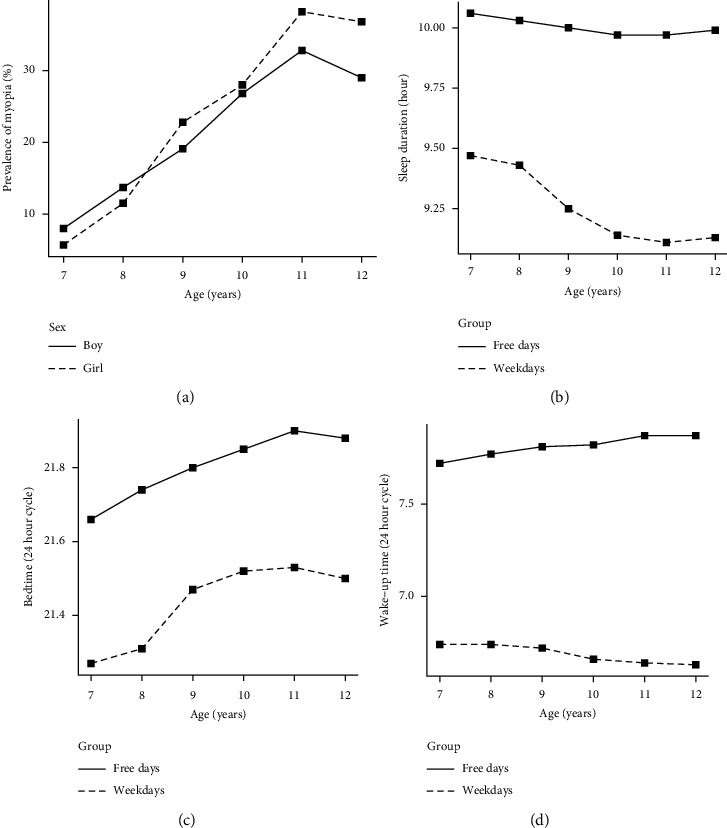
Prevalence of myopia and sleep traits in 7–12-year-old children. Changes in prevalence of myopia (a), sleep duration (b), bedtime (c), and wake-up time (d) according to age.

The study sample included 10,142 children (5305 boys, 52.3%), aged 9.12 years (SD = 1.47, ranging from 7 to 12 years old), and the prevalence of myopia was 21.2%. The sample characteristics are shown in Table 1. Compared with those without myopia, individuals with myopia were older and more likely to be girls, they tend to have less outdoor activity and TV time, higher homework time, and digital screen exposure, and their father and mother had higher education levels and income (all *P* < 0.05). Meanwhile, they fall asleep and wake up relatively later and tend to have shorter night's sleep and MSFsc. After propensity score matching, all covariates were balanced, and the differences in sleep duration, sleep midpoint, and wake-up time on weekdays between the two groups were still kept (all *P* < 0.05).  Figure 1 shows the prevalence of myopia and sleep features in children aged 7–12 years.

### 3.2. The Associations between Sleep Duration and Myopia

It was observed that the increased SDW was related to a declining risk of myopia after adjusting for possible confounders. The ORs (vs. <9 hours/day) were 0.87 (95% CI: 0.77 to 0.98) for 9-10 hours/day and 0.77 (95% CI: 0.64 to 0.92) for ≥10 hours/day (both *P* < 0.05, *P* for trend< 0.01). After propensity score matching, a similar tendency was also observed. The OR was 0.76 (95% CI: 0.62 to 0.95) for ≥10 hours/day (*P* < 0.05, *P* for trend< 0.05) (Table 2).

### 3.3. The Associations between Sleep Timing and Myopia

The delay in bedtime and wake-up time on weekdays was related to an increased risk of myopia after adjusting for possible confounders, in which the ORs of bedtime (vs. before 9 pm) were 1.46 (95% CI: 1.16 to 1.83) for 9 pm to 9:30 pm, 1.51 (95% CI: 1.18 to 1.93) for 9:30 pm to 10 pm, and 2.08 (95% CI: 1.57 to 2.77) for 10 pm and after and the OR of wake-up time (vs. before 6:30 am) was 1.36 (95% CI: 1.16 to 1.60) for 7 am and after (both *P* < 0.05, *P* for trend< 0.001). After propensity score matching, a similar tendency was also observed. In addition, the delay of wake-up time on free days increased the risk of myopia, with the OR (vs. before 7:30 am) of 1.17 (95% CI: 1.01 to 1.35) for 8 am and after (*P* < 0.05, *P* for trend< 0.05) (Table 3).

### 3.4. The Associations between Sleep Consistency, Chronotype, and Myopia

Compared with the no catch-up group (≤0 hour), after adjusting for possible confounders, the ORs of CUS duration were 1.15 (95% CI: 1.00 to 1.32) for 0 to 1 hour and 1.42 (95% CI: 1.23 to 1.64) for ≥1 hours (both *P* < 0.05, *P* for trend< 0.001). Concerning social jetlag, we found that it was associated with lower odds of myopia (OR = 0.67, 95% CI: 0.58 to 0.78). As for chronotype, the ORs (vs. morning chronotype) were 1.28 (95% CI: 1.11 to 1.46) for intermediate chronotype and 1.42 (95% CI: 1.21 to 1.68) for evening chronotype (both *P* < 0.05, *P* for trend< 0.001). After propensity score matching, a similar tendency was also observed (Table 4).

## 4. Discussion

Based on large cross-sectional data, the present study explored which sleep variables were involved in higher risk of school myopia. We found workday sleep duration and sleep timing were correlated with myopia in a dose-dependent pattern to some extent, in which longer sleep duration was associated with decreased risk of myopia, while later bedtime, wake-up time, and sleep midpoint increased the risk. Moreover, this study, for the first time, assessed the associations of sleep consistency (CUS and social jetlag) and chronotype with myopia, showing longer CUS duration and later chronotype were positively correlated with myopia, while social jetlag was the opposite. These findings provided an enriched insight into the relationship between sleep and childhood myopia.

### 4.1. Sleep Duration with Myopia

The linkage between sleep duration and myopia among school-age children and adolescents has been inconsistent in the literature. A Korean study found the adjusted OR for myopia was decreased in those with >9 hours of sleep (OR: 0.59, 95% CI: 0.38–0.93) than in those with <5 hours of sleep among adolescents aged 12 to 19 years, and there existed a dose‐effect relationship [17]. This finding was also supported by another Chinese study aimed at school-aged children [34]. A study carried out in Japan also demonstrated that shorter sleep duration in children aged 10 to 19 years was significantly correlated with high myopia [35]. However, another study including 1831 Chinese students aged 11–18 years thought that myopia was not statistically associated with sleep duration, whenever in the semester and holidays [26]. Two recent cohort studies also provided inconsistent results; in a 4-year follow-up of Chinese children's study, no significant association was found for sleep duration with myopia progression and axial elongation for the children and the association with axial elongation was only of borderline significance in girls [15]. In another French cohort study, a U-shaped association was observed between sleep duration at age 2 and eyeglass prescription at age 5 [14]. Different ages as well as the lack of controlling for confounding variables in these studies may contribute to the conflicting results. In our study, after adjusting for possible confounders, whether or not propensity scoring, longer sleep duration on weekdays was always associated with a lower risk of childhood myopia, while the trend has not yet been observed on free days. Since Chinese school students were always with cumbersome academic burdens, compared to free days, sleep duration on weekdays is more representative of children's true status, and this could explain the different results between weekdays and free days.

### 4.2. Sleep Timing with Myopia

A sole focus on sleep duration may oversimplify the importance of sleep as a time use behavior, and other aspects of sleep, like bedtime, wake-up time, and sleep midpoint, should be noted. We found that the later bedtime and wake-up time on weekdays, as well as later wake-up time on free days, increased the risk of myopia, and as MSW raised, the risk increased. In a Chinese follow-up study aimed at school-aged children, sleeping late (21 : 30 and after) was a risk factor for myopia prevalence at baseline (OR: 1.55, 95% CI: 1.02 to 2.34) and 2-year myopia incidence (OR: 1.45, 95% CI: 1.05 to 2.00) [16]. Another cross-sectional study also found myopia was positively associated with a later bedtime during holidays [26]. In a French cohort study, the later midpoint of sleep and bedtime at age 2 were associated with the increased risk of eyeglass prescription at age 5 [14]. Overall, most existing evidence believed that there was a link between sleep timing and myopia and our research further proved this linkage.

### 4.3. Sleep Consistency and Chronotype with Myopia

Sleep consistency and chronotype are closely linked to individual health [18, 19],but no research has focused on the impacts on myopia. We found longer CUS duration and later chronotype were positively correlated with myopia, while social jetlag is the opposite. CUS is assumed to be an indicator of compensation for sleep debt; more CUS is indirect evidence of sleep deprivation and represents an irregular sleep pattern [20, 21]. Children and youths with longer CUS duration may be more likely to have poorer academic performance, more depressive symptoms, and even increased risks of obesity and executive dysfunction [20, 36]. We further demonstrated the association of CUS duration with myopia.

Contrary to CUS, a negative association was found between social jetlag and myopia, which seems to contradict the usual perception. Differences in sleep timing (e.g., bedtime, rise time, and the midpoint of sleep) between workday and free day simulate the effects of jet lag, defined as “social jetlag” [36], reflecting the discrepancy between social and circadian rhythms [22]. The existing evidence indicates that, among adolescents, high social jetlag is associated with behavioral problems and obesity [37, 38], which are not observed among children [39]. There is a shift in biological preference for later sleep timing and higher social jetlag during adolescence; by contrast, the sleep pattern is relatively stable and regular during school age [40]. Our study revealed a far less than 1 hour's jetlag of sleep midpoint between weekdays and free days, suggesting social jetlag is quite mild in our sampled school-aged children. Similar to our finding, the most recent study did not establish a relationship between social jetlag and negative health outcome [39]. In summary, the methods and thresholds for characterizing biologically social jetlag in children are not well understood, and further exploration is needed.

Chronotype refers to the relation of an individual's endogenous circadian clock to the 24-hour day, and it represents the preferences with regard to the timing for engaging in sleep and other activities [23, 24]. Chronotype is usually categorized into three types: morning-type, intermediate-type, and evening-type [29]. Previous studies believed that compared with the morning chronotype, evening chronotype could lead to psychological, behavioral, and metabolic problems among children [19, 24, 41]. This study, for the first time, assessed the impact of chronotype on school myopia, demonstrating that evening and intermediate chronotypes, by comparison with morning chronotype, were positively correlated with myopia.

### 4.4. Potential Biological Mechanism

The potential biological mechanism research mainly focuses on circadian rhythm [12]. Normal circadian rhythms are essential to the development of the human eye [42], and the disruption of these rhythms by inadequate sleep duration and irregular sleep rhythms may interfere with regulatory mechanisms controlling eye growth which underlie the emmetropization process, leading to refractive errors [12, 43]. In the 1960s, an animal study found elongated eyes and flat corneas in chicks exposed to continuous light, which led to the hypothesis that the absence of normal ocular diurnal rhythms results in abnormal eye growth and refractive error development [43]. Hereafter, several ocular biometry parameters were found to exhibit diurnal rhythms in animal studies [43, 44]. Similar to findings in different animal eyes, the axial length of the human eye is typically the longest during the midday and shortest at night, with an average magnitude of diurnal variation of around 25 to 45 *μ*m, equating to approximately 0.06 to 0.11 D [12, 45]. Diurnal fluctuations in choroidal thickness are in approximate antiphase to that of axial length, having a mean diurnal amplitude of about 30 *μ*m, resulting in a small refractive effect of about 0.18 D [45]. Circadian rhythm disorder of these biometry parameters has important implications in the development of refractive errors, especially myopia [12, 43]. A recent meta-analysis of a genome-wide association study (GWAS) further strengthened the evidence supporting the role of circadian rhythm in the development of myopia, in which genetic factors regulating circadian rhythm are identified to participate in the development of myopia and refractive error [46]. Alternatively, systemic circadian rhythms are primarily regulated by the hypothalamic suprachiasmatic nucleus (SCN) in the brain which also controls the circadian release of dopamine [15, 47, 48].A previous study found that sleep deprivation downregulated dopamine D2 receptors in the ventral striatum in the human brain [47], which may lead to less activation of retinal dopaminergic pathways, resulting in refractive errors [15]. Furthermore, as an indicator of circadian rhythm, melatonin release increases at night and decreases throughout the daytime under the guidance of the hypothalamic suprachiasmatic nucleus [49]. Dopamine and melatonin together organize the retinal circadian rhythmicity through dopamine D-4 and MT1 receptors, while the former is mainly synthesized during the daytime [50]. Studies have revealed opposite roles of dopamine and melatonin in regulating the physiological functions of the eye and dopamine can reduce the expression of the melatonin synthesis enzyme AANAT, resulting in the reduction of melatonin synthesis [50]. A number of animal models have provided evidence that myopia is related to increased levels of melatonin receptor expression [42, 51, 52]. Melatonin and its analogs, when applied systemically or intravitreally, have been found to cause significant alterations in ocular development in chickens [51, 53, 54]. Taken together, melatonin should play an important role in refractive development.

### 4.5. Limitations

Several limitations should be noticed. Firstly, our cross-sectional study was incapable of explaining the causal links, and the relationship is worth further exploration. In addition, although our study included a large number of confounding factors, we did not get the information on parental myopia, while a few studies believed that genetic factors alone cannot explain the high incidence of school myopia, and recent GWAS found associated genetic variants only explain 18.4% of heritability in refractive errors [8, 46]. Finally, the results we present were according to self-reported data; however, myopia was determined by the doctor's diagnosis, and sleep characteristics were based on validated questionnaires. Future research can build on our study with more biophysical and myopia and more objective measurements of sleep.

## 5. Conclusions

In conclusion, we found that there were obvious relationships between multiple dimensions of sleep and childhood myopia. In addition to sleep duration and timing, sleep consistency, and chronotype were also strictly related to myopia. These findings provided an enriched insight into the relationship between sleep and childhood myopia. Given that childhood, the critical period for the control of myopia, is vulnerable to disrupted sleep, particular emphasis should be placed on sleep health in the management of myopia.

## Figures and Tables

**Table 1:** Baseline characteristics of the total sample and propensity score-matched sample.

	Total sample (*N* = 10142)			Propensity 1 : 1 matching (*N* = 4288)		
	Control	Case	*P*	Control	Case	*P*
	(*n* = 7995)	(*n* = 2147)		(*n* = 2144)	(*n* = 2144)	
Demographic factors						
Age (years)	8.93 (1.46)	9.83 (1.30)	**7.33E−142**	9.84 (1.34)	9.82 (1.30)	0.691
Sex (Boy, %)	4223 (52.8)	1082 (50.4)	**0.046**	1084 (50.6)	1082 (50.5)	0.951
BMI (kg/m^2^)	17.4 (3.29)	17.6 (3.37)	**0.025**	17.57 (3.34)	17.60(3.37)	0.785
Fathers' education level (high school or higher, %)	6763 (84.6)	1943 (90.5)	**3.13E−12**	1927 (89.9)	1940 (90.5)	0.505
Mothers' education level (high school or higher, %)	6456 (80.8)	1885 (87.8)	**3.31E−14**	1892 (88.2)	1882 (87.8)	0.638
Per capita income (≥ 6000 ¥/month, %)	4983 (62.3)	1475 (68.7)	**4.97E−08**	1469 (68.5)	1472 (68.7)	0.921
Behavioral factors						
Outdoor time on weekdays (≥2 hours/day, %)	421 (5.3)	85 (4.0)	**0.014**	106 (4.9)	85 (4.0)	0.120
Outdoor time on free days (≥2 hours/day, %)	1685 (21.1)	372 (17.3)	**1.25E−04**	396 (18.5)	372 (17.4)	0.339
Homework time on weekdays (≥3 hours/day, %)	627 (7.8)	211 (9.8)	**0.003**	202 (9.4)	211 (9.8)	0.641
Homework time on free days (≥3 hours/day, %)	1718 (21.5)	607 (28.3)	**3.15E−11**	583 (27.2)	606 (28.3)	0.433
TV time (≥2 hour/day, %)	249 (3.1)	47 (2.2)	**0.024**	55 (2.6)	47 (2.2)	0.423
Digital screen exposure (≥10 times/week, %)	713 (8.9)	238 (11.1)	**0.002**	232 (10.8)	237 (11.1)	0.807
Dietary factors						
Fried food (≥1 time/month, %)	1170 (14.6)	330 (15.4)	0.394	356 (16.6)	330 (15.4)	0.279
Carbonated drinks (≥1 time/month, %)	4789 (59.9)	1314 (61.2)	0.274	1350 (63.0)	1312 (61.2)	0.232
Vitamin D (≥5 times/week, %)	1159 (14.5)	315 (14.7)	0.838	312 (14.6)	315 (14.7)	0.897
Sleep variables						
Sleep duration						
SDW (h)	9.30 (0.60)	9.16 (0.58)	**6.25E−24**	9.19 (0.61)	9.16 (0.58)	**0.045**
SDF (h)	10.01 (0.80)	9.98 (0.77)	**0.049**	9.99 (0.81)	9.98 (0.77)	0.599
Average sleep duration (h)	9.51 (0.54)	9.39 (0.52)	**8.76E−19**	9.42 (0.55)	9.39 (0.52)	0.071
Sleep timing						
Wake-up time on weekdays (hh: mm)	21:23 (35)	21:34 (34)	**3.82E−35**	21:18 (35)	21:34 (34)	**2.70E−04**
Bedtime on weekdays (hh: mm)	06:41 (23）	06:43 (23)	**0.004**	06:41 (24)	06:43 (23)	**0.021**
Wake-up time on free days (hh: mm)	21:46 (38）	21:53 (36)	**6.33E−13**	21:51 (38)	21:53 (36)	0.126
Bedtime on free days (hh: mm)	07:42 (49)	07:52 (48)	**2.91E−04**	07:50 (49)	07:52 (48)	0.517
MSW (hh: mm)	02:02 (23)	02:08 (23)	**5.50E−26**	02:05 (23)	02:08 (23)	**1.30E−04**
MSF (hh: mm)	02:47 (36)	02:52 (36)	**9.59E−10**	02:51 (36)	02:52 (36)	0.223
Sleep consistency and chronotype						
CUS duration (h)	0.71 (0.85)	0.82 (0.83)	**1.33E−07**	0.80 (0.85)	0.82 (0.83)	0.358
Social jetlag (h)	0.76 (0.50)	0.75 (0.49)	0.304	0.77 (0.49)	0.75 (0.49)	0.069
MSFsc (h)	2.50 (0.55)	2.56 (0.52)	**4.00E−06**	2.54 (0.54)	2.56 (0.52)	0.256
Sleep disorders (Yes, %)	6421 (80.3)	1692 (78.8)	0.122	1682 (78.5)	1690 (78.8)	0.766

Continuous variables are presented with mean and standard deviation, categorical variables are presented with frequency and percentage of columns. SDW: sleep duration on workdays; SDF: sleep duration on free days; MSW: sleep midpoint on workdays; MSF: sleep midpoint on free days; CUS: weekend catch-up sleep; ¥: China's currency (yuan).

**Table 2:** Associations between sleep duration on workdays and free days with myopia.

	Total sample (*N* = 10142)					Propensity 1 : 1 matching (*N* = 4288)				
	Case (*N*)	Crude model		Adjusted model		Case (*N*)	Crude model		Adjusted model	
		OR (95% CI)	*P*	OR (95% CI)	*P*		OR (95% CI)	*P*	OR (95% CI)	*P*
SDW										
<9 (hours/day)	600 (2134)	Ref.		Ref.		599 (1146)	Ref.		Ref.	
9-10 (hours/day)	1302 (6398)	0.66 (0.58,0.73)	**<0.001**	0.87 (0.77,0.98)	**<0.05**	1300 (2607)	0.91 (0.79,1.04)	0.175	0.90 (0.78, 1.04)	0.144
≥10 (hours/day)	245 (1610)	0.46 (0.39,0.54)	**<0.001**	0.77 (0.64,0.92)	**<0.01**	245 (535)	0.77 (0.63,0.95)	**<0.05**	0.76 (0.62, 0.95)	**<0.05**
*P* for trend			**<0.001**		**0.002**			**<0.05**		**<0.05**
SDF										
<9 (hours/day)	90 (380)	Ref		Ref.		90 (184)	Ref.		Ref.	
9-10 (hours/day)	694 (3178)	0.90 (0.70,1.16)	0.412	1.06 (0.81,1.38)	0.672	693 (1403)	1.02 (0.75,1.39)	0.902	1.01 (0.74,1.38)	0.938
≥10 (hours/day)	1363 (6584)	0.84 (0.66,1.07)	0.165	1.06 (0.82,1.36)	0.676	1361 (2701)	1.06 (0.79,1.43)	0.699	1.05 (0.78,1.42)	0.754
*P* for trend			0.077		0.766			0.533		0.595

OR: odds ratio; CI, confidence interval; SDW: sleep duration on workdays; SDF: sleep duration on free days. Crude model: calculated by the univariate logistic regression model. Adjusted model: based on crude model, adjusted for age, gender, BMI, father and mothers' education level, per capita income, outdoor time on weekdays/free days, homework time on weekdays/free days, TV time, electronic product exposure, fried food, carbonated drinks, vitamin D, sleep disorders.

**Table 3:** Associations between sleep timing on workdays and free days with myopia.

	Total sample (*N* = 10142)					Propensity 1 : 1 matching (*N* = 4288)				
	Case (*N*)	Crude model		Adjusted model		Case (*N*)	Crude model		Adjusted model	
		OR (95% CI)	*P*	OR (95% CI)	*P*		OR (95% CI)	*P*	OR (95% CI)	*P*
Bedtime on weekdays										
Before 9 pm	122 (1008)	Ref.		Ref.		122 (290)	Ref.		Ref.	
9 pm to 9:30 pm	592 (3275)	1.60 (1.30,1.98)	**<0.001**	1.46 (1.16,1.83)	**<0.01**	592 (1212)	1.32 (1.02,1.70)	**<0.05**	1.42 (1.08,1.86)	**<0.05**
9:30 pm to 10 pm	653 (3154)	1.90 (1.54,2.33)	**<0.001**	1.51 (1.18,1.93)	**<0.01**	653 (1349)	1.29 (1.00,1.67)	**<0.05**	1.47 (1.10, 1.98)	**<0.05**
10 pm and after	780 (2705)	2.94 (2.39,3.62)	**<0.001**	2.08 (1.57,2.77)	**<0.001**	777 (1437)	1.62 (1.26,2.09)	**<0.001**	2.00 (1.42, 2.82)	**<0.001**
*P* for trend			**<0.001**		**<0.001**			**<0.001**		**<0.001**

Wake-up time on weekdays										
Before 6:30 am	364 (1798)	Ref.		Ref.		363 (777)	Ref.		Ref.	
6:30 am to 7 am	953 (4674)	1.01 (0.88,1.16)	0.897	1.15 (0.99,1.32)	0.069	953 (1895)	1.15 (0.98,1.36)	0.094	1.23 (1.03,1.46)	**<0.05**
7 am and after	830 (3670)	1.15 (1.00,1.32)	**<0.05**	1.36 (1.16,1.60)	**<0.001**	828 (1616)	1.20 (1.01,1.42)	**<0.05**	1.35 (1.12,1.64)	**<0.01**
*P* for trend			**<0.014**		**<0.001**			0.061		**<0.01**

Bedtime on free days										
Before 10 pm	798 (4338)	Ref.		Ref.		798 (1603)	Ref.		Ref.	
10 pm to 10:30 pm	881 (3963)	1.27 (1.14,1.41)	**<0.001**	1.01 (0.90,1.13)	0.892	880 (1780)	0.99 (0.86,1.13)	0.842	0.99 (0.86,1.14)	0.870
10:30 pm to 11 pm	306 (1196)	1.53 (1.31,1.77)	**<0.001**	1.06 (0.90,1.26)	0.469	305 (586)	1.10 (0.91,1.32)	0.348	1.10 (0.90,1.35)	0.341
11 pm and after	162 (645)	1.49 (1.23,1.81)	**<0.001**	0.99 (0.80,1.23)	0.918	161 (319)	1.03 (0.81,1.31)	0.822	1.05 (0.81,1.36)	0.726
*P* for trend			**<0.001**		0.750			0.528		0.472

Wake-up time on free days										
Before 7:30 am	543 (2878)	Ref.		Ref.		542 (1122)	Ref.		Ref.	
7:30 am to 8 am	392 (1883)	1.13 (0.98,1.31)	0.098	1.11 (0.95,1.29)	0.207	392 (774)	1.10 (0.91,1.32)	0.317	1.13 (0.94,1.36)	0.206
8 am and after	1212 (5381)	1.25 (1.12,1.40)	**<0.001**	1.17 (1.01,1.35)	**<0.05**	1210 (2392)	1.10 (0.95,1.26)	0.208	1.19 (0.99,1.42)	0.053
*P* for trend			**<0.001**		**<0.05**			0.239		0.056

OR: odds ratio; CI, confidence interval. Crude model: calculated by the univariate logistic regression model. Adjusted model: based on crude model, adjusted for age, gender, BMI, father and mothers' education level, per capita income, outdoor time on weekdays/free days, homework time on weekdays/free days, TV time, electronic product exposure, fried food, carbonated drinks, vitamin D, sleep disorders, and SDW or SDF.

**Table 4:** Associations between sleep consistency and chronotype with myopia.

	Total sample (*N* = 10142)					Propensity 1 : 1 matching (*N* = 4288)				
	Case (*N*)	Crude model		Adjusted model		Case (*N*)	Crude model		Adjusted model	
		OR (95% CI)	*P*	OR (95% CI)	*P*		OR (95% CI)	*P*	OR (95% CI)	*P*
Sleep consistency										
Social jetlag	–	0.95 (0.86,1.05)	0.304	0.67 (0.58,0.78)	**<0.001**	–	0.89 (0.79,1.01)	0.069	0.73 (0.61,0.87)	**<0.001**
CUS duration										
No catch-up (≤0 hours)	466 (2597)	Ref.		Ref.		465 (968)	Ref.		Ref.	Ref.
0-1 hour	703 (3465)	1.16 (1.02,1.33)	**<0.05**	1.15 (1.00,1.32)	**<0.05**	702 (1431)	1.04 (0.89,1.23)	0.624	1.05 (0.89,1.24)	0.558
≥1 hours	978 (4080)	1.44 (1.27,1.63)	**<0.001**	1.42 (1.23,1.64)	**<0.01**	977 (1889)	1.16 (0.99,1.35)	0.062	1.31 (1.10,1.56)	**<0.01**
*P* for trend			**<0.001**		**<0.001**			**<0.05**		**<0.001**
Chronotype										
Morning	619 (3391)	Ref.		Ref.		619 (1295)	Ref.		Ref.	Ref.
Intermediate	743 (3266)	1.32 (1.17,1.49)	**<0.001**	1.28 (1.11,1.46)	**<0.001**	742 (1418)	1.20 (1.03,1.39)	**<0.05**	1.31 (1.11,1.54)	**<0.01**
Evening	785 (3485)	1.30 (1.16,1.47)	**<0.001**	1.42 (1.21,1.68)	**<0.001**	783 (1575)	1.08 (0.93,1.25)	0.307	1.38 (1.13,1.68)	**<0.01**

OR: odds ratio; CI: confidence interval; CUS: weekend catch-up sleep. Crude model: calculated by the univariate logistic regression model. Adjusted model: based on crude model, adjusted for age, gender, BMI, father and mothers' education level, per capita income, outdoor time on weekdays/free days, homework time on weekdays/free days, TV time, electronic product exposure, fried food, carbonated drinks, vitamin D, sleep disorders, average sleep duration, CUS duration, social jetlag, and chronotype.

## Data Availability

The datasets used and/or analyzed during the current study are available from the corresponding author on reasonable request.
